# Life expectancy in rare histological prostate cancer subtypes

**DOI:** 10.1002/ijc.35323

**Published:** 2024-12-30

**Authors:** Carolin Siech, Mario de Angelis, Letizia Maria Ippolita Jannello, Francesco Di Bello, Natali Rodriguez Peñaranda, Jordan A. Goyal, Zhe Tian, Fred Saad, Shahrokh F. Shariat, Stefano Puliatti, Nicola Longo, Ottavio de Cobelli, Alberto Briganti, Mike Wenzel, Philipp Mandel, Luis A. Kluth, Felix K. H. Chun, Pierre I. Karakiewicz

**Affiliations:** ^1^ Cancer Prognostics and Health Outcomes Unit, Division of Urology University of Montréal Health Center Montréal Québec Canada; ^2^ Department of Urology Goethe University Frankfurt, University Hospital Frankfurt am Main Germany; ^3^ Division of Experimental Oncology/Unit of Urology URI, IRCCS Ospedale San Raffaele Milan Italy; ^4^ Vita‐Salute San Raffaele University Milan Italy; ^5^ Department of Urology IEO European Institute of Oncology, IRCCS Milan Italy; ^6^ Università degli Studi di Milano Milan Italy; ^7^ Department of Neuroscience, Science of Reproduction and Odontostomatology University of Naples Federico II Naples Italy; ^8^ Department of Urology, AOU di Modena University of Modena and Reggio Emilia Modena Italy; ^9^ Department of Urology Comprehensive Cancer Center, Medical University of Vienna Vienna Austria; ^10^ Department of Urology Weill Cornell Medical College New York New York USA; ^11^ Department of Urology University of Texas Southwestern Medical Center Dallas Texas USA; ^12^ Hourani Center for Applied Scientific Research, Al‐Ahliyya Amman University Amman Jordan; ^13^ Department of Oncology and Haemato‐Oncology Università degli Studi di Milano Milan Italy

**Keywords:** overall survival, rare diseases, SEER, social security administration life tables, variant histology

## Abstract

Survival differences in rare histological prostate cancer (PCa) subtypes relative to age‐matched population‐based controls are unknown. Within Surveillance, Epidemiology, and End Results database (2004–2020), newly diagnosed (2004–2015) PCa patients were identified. Relying on the Social Security Administration Life Tables (2004–2020) with 5 years of follow‐up, age‐matched population‐based controls (Monte Carlo simulation) were simulated for each patient. Kaplan–Meier analyses addressed survival rates. Of 582,220 patients, 580,368 (99.68%) harbored acinar, 867 (0.15%) ductal, 534 (0.09%) neuroendocrine, 368 (0.07%) mucinous, and 83 (0.01%) signet ring cell carcinoma. The metastatic stage was most prevalent in neuroendocrine (62%). In the localized stage, the overall survival difference at 5 years of follow‐up was greatest in neuroendocrine (22% vs. 72%), signet ring cell (78% vs. 84%), and ductal carcinoma (71% vs. 77%). In the locally advanced stage, overall survival difference was greatest in neuroendocrine (16% vs. 79%), signet ring cell (75% vs. 91%), ductal (78% vs. 84%), and mucinous carcinoma (84% vs. 90%). In the metastatic stage, the overall survival difference was greatest in neuroendocrine (3% vs. 81%), mucinous (26% vs. 84%), and acinar carcinoma (27% vs. 85%). Regardless of stage, neuroendocrine carcinoma patients exhibit the least favorable life expectancy compared with population‐based controls. Conversely, all other rare histological PCa subtypes do not meaningfully affect life expectancy in localized or locally advanced stages, except for locally advanced signet ring cell adenocarcinoma.

## What's New?

Rare histological subtypes of prostate cancer often are associated with relatively poor clinical outcomes. The extent to which these subtypes impact patients' life expectancy, however, remains largely unknown. Here, using data from Surveillance, Epidemiology, and End Results (SEER 2004–2020) and the United States Social Security Administration Life Tables, the life expectancy of patients diagnosed with a rare prostate cancer subtype was compared with that of population‐matched controls. Life expectancy was found to be least favorable for neuroendocrine carcinoma, followed by locally advanced signet ring cell adenocarcinoma. Other rare subtypes had little impact on life expectancy in localized or locally advanced stages.

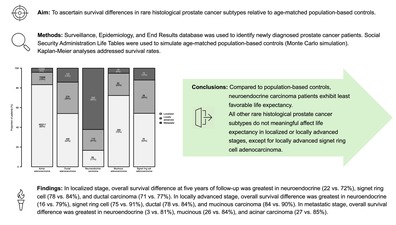

## 1 INTRODUCTION

Acinar adenocarcinoma is the dominant histology of newly diagnosed prostate cancer (PCa).^1^, ^2^, ^3^, ^4^ Rare histological PCa subtypes include ductal, neuroendocrine, mucinous, or signet ring cell carcinoma.^1^, ^2^, ^3^, ^4^, ^5^, ^6^, ^7^ Previous reports describe poor clinical outcomes in the majority of these histological PCa subtypes compared with acinar adenocarcinoma.^1^, ^2^, ^3^, ^4^, ^5^, ^8^ However, these survival analyses compared patients with different histological subtypes with each other but did not quantify to what extent such diagnoses undermine patients' life expectancy relative to that of their age‐matched male population‐based controls. Moreover, these reports did not take stage‐specific considerations into account.

We addressed this knowledge gap. Specifically, we hypothesized that pronounced differences in overall survival (OS) rates exist between patients with some histological PCa subtypes and their population‐based controls, especially in locally advanced and metastatic stages. Conversely, we posited that differences in OS relative to age‐matched male population‐based controls are less pronounced, if present at all, in patients with localized stage across all examined histological PCa subtypes. To test these hypotheses, we relied on both the Surveillance, Epidemiology, and End Results (SEER 2004–2020) database to identify PCa patients and the United States Social Security Administration (SSA) Life Tables to simulate age‐matched male population‐based controls.^9^

## 2 MATERIALS AND METHODS

### 2.1 Data source and study population

Within the SEER database (2004–2020; https://seer.cancer.gov/data/) that provides cancer statistics covering ~47.9% of the United States population,^10^ we identified newly diagnosed (2004–2015) and histologically confirmed PCa (International Classification of Diseases [ICD‐10] site code C61). Specifically, we included the following rare histological subtypes: ductal (International Classification of Disease for Oncology [ICD‐O‐3] codes 8201/3, 8260/3, 8380/3, 8500/3, 8501/3, 8503/3, 8521/3, 8523/3, and 8552/3), neuroendocrine (ICD‐O‐3 codes 8013/3, 8020/3, 8041/3, 8045/3, 8240/3, 8244/3, and 8246/3), mucinous (ICD‐O‐3 codes 8480/3, and 8481/3), and signet ring cell carcinoma (ICD‐O‐3 codes 8490/3). Patients with acinar adenocarcinoma (ICD‐O‐3 codes 8140/3, 8550/3, and 8551/3) were included for comparison. All other histologic subtypes were excluded. Only patients aged at least 18 years with known vital status, known cause of death, and known stage were included. Autopsy‐ or death certificate‐only cases were excluded.

### 2.2 Study endpoints

The primary study endpoint in PCa patients represented observed OS, defined as survival after consideration of all causes of death. Conversely, life expectancy was simulated and quantified in age‐matched male population‐based controls based on Life Tables data.

### 2.3 Statistical analyses

First, baseline characteristics of PCa patients were tabulated. Descriptive statistics of PCa patients included medians and interquartile ranges (IQR) for continuously coded variables and frequencies and proportions for categorical variables. Second, to estimate life expectancy in age‐matched male population‐based controls, we relied on a Monte Carlo simulation. Here, a one‐to‐one matching process was applied for each acinar adenocarcinoma patient.^11^, ^12^, ^13^, ^14^ Due to the rarity of other histological PCa subtypes, a one‐to‐four matching was applied for these patients. Additionally, a Markov chain representing the natural progression of age according to United States SSA Life Tables data was defined for each control.^9^ Thus, simulated age‐matched male population‐based controls will henceforth be referred to as “controls”. Third, OS data for cases and controls were displayed in Kaplan–Meier plots. All analyses were performed in stage‐specific fashion (localized vs. locally advanced vs. metastatic stage) within each histological PCa subtype (acinar vs. ductal vs. neuroendocrine vs. mucinous vs. signet ring cell carcinoma). All statistical tests were two sided with a level of significance set at *p* < .05. R software environment was used for statistical computing and graphics (R version 4.3.2; R Foundation for Statistical Computing, Vienna, Austria).^15^

## 3 RESULTS

### 3.1 Descriptive characteristics of PCa patients

**FIGURE 1:**
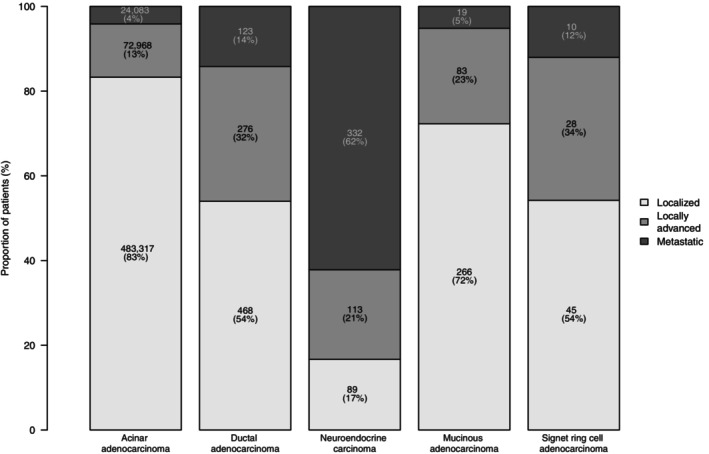
Stage distribution of newly diagnosed patients with acinar adenocarcinoma and rare histological PCa subtypes within the Surveillance, Epidemiology, and End Results database.

Within the SEER database (2004–2020), we identified 582,220 PCa patients newly diagnosed between 2004 and 2015. Of these, 580,368 (99.68%) harbored acinar, 867 (0.15%) ductal, 534 (0.09%) neuroendocrine, 368 (0.07%) mucinous, and 83 (0.01%) signet ring cell carcinoma (Table 1). In histologic subtype specific assessment, median age was 63 years (IQR 56–69 years) in mucinous, 66 years (IQR 60–73 years) in acinar, 67 years (IQR 63–73 years) in signet ring cell, 69 years (IQR 62–77 years) in ductal, and 71 years (IQR 63–79 years) in neuroendocrine carcinoma. Metastatic stage was most prevalent in neuroendocrine carcinoma (62%), followed by ductal (14%), signet ring cell (12%), mucinous (5%), and acinar adenocarcinoma (4%; Figure 1).

**TABLE 1:** Descriptive characteristics of newly diagnosed patients with acinar adenocarcinoma and rare histological PCa subtypes within the Surveillance, Epidemiology, and End Results database.

Characteristic	Acinar adenocarcinoma, *n* = 580,368 (99.68%)	Ductal adenocarcinoma, *n* = 867 (0.15%)	Neuroendocrine carcinoma, *n* = 534 (0.09%)	Mucinous adenocarcinoma, *n* = 368 (0.07%)	Signet ring cell adenocarcinoma, *n* = 83 (0.01%)
Age (in years)^a^	66 (60, 73)	69 (62, 77)	71 (63, 79)	63 (56, 69)	67 (63, 73)
Stage^b^					
Localized	483,317 (83%)	468 (54%)	89 (17%)	266 (72%)	45 (54%)
Regional	72,968 (13%)	276 (32%)	113 (21%)	83 (23%)	28 (34%)
Distant	24,083 (4%)	123 (14%)	332 (62%)	19 (5%)	10 (12%)

^a^ Median (IQR).

^b^ *n* (%).

### 3.2 OS in localized stage PCa patients versus controls

**FIGURE 2:**
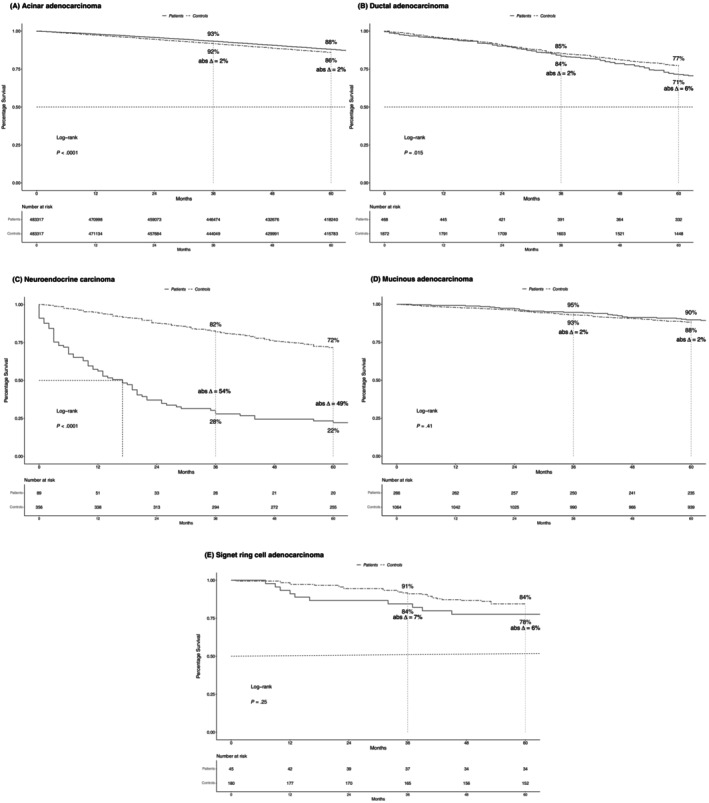
Kaplan–Meier plots comparing overall survival (OS) of patients with localized stage (A) acinar, (B) ductal, (C), neuroendocrine, (D) mucinous, (E) signet ring cell carcinoma of the prostate to their simulated age‐matched male population‐based controls. Abs Δ, absolute difference; OS, overall survival.

In localized stage, 5‐year OS rates were 22% in neuroendocrine carcinoma, 71% in ductal, 78% in signet ring cell, 88% in acinar, and 90% in mucinous adenocarcinoma patients (Figure 2A–E). In controls that were simulated to match localized stage PCa patients, corresponding OS rates were 72%, 77%, 84%, 86%, and 88%, respectively. The resulting differences in 5‐year OS rates between PCa patients versus controls (Δ) were −49% for neuroendocrine versus −6% for ductal and signet ring cell versus +2% for acinar and mucinous carcinoma.

### 3.3 OS in locally advanced stage PCa patients versus controls

**FIGURE 3:**
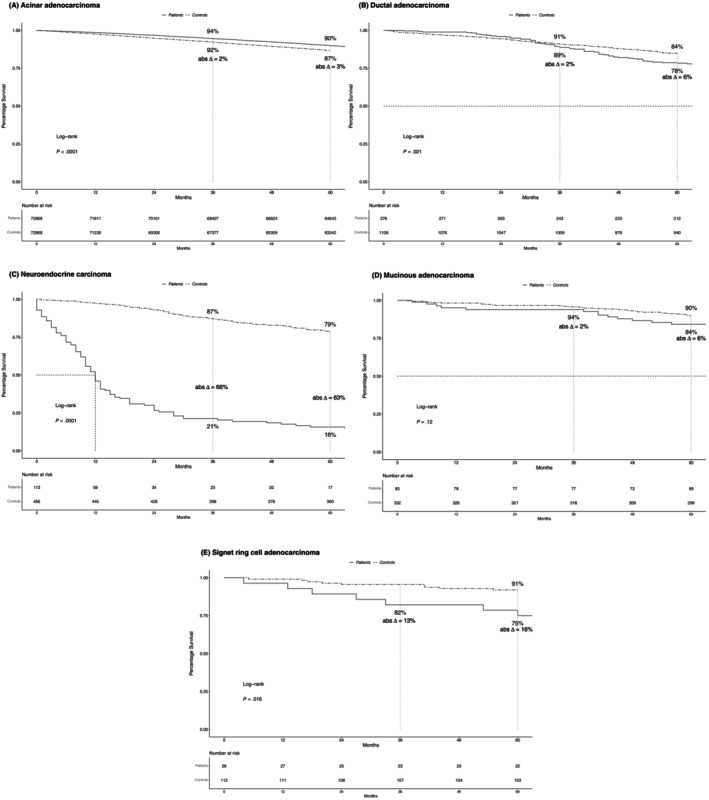
Kaplan–Meier plots comparing overall survival (OS) of patients with locally advanced stage (A) acinar, (B) ductal, (C), neuroendocrine, (D) mucinous, (E) signet ring cell carcinoma of the prostate to their simulated age‐matched male population‐based controls. Abs Δ, absolute difference; OS, overall survival.

In locally advanced stage, 5‐year OS rates were 16% in neuroendocrine carcinoma, 75% in signet ring cell, 78% in ductal, 84% in mucinous, and 90% in acinar adenocarcinoma patients (Figure 3A–E). In controls that were simulated to match locally advanced stage PCa patients, corresponding OS rates were 79%, 91%, 84%, 90%, and 87%, respectively. The resulting differences in 5‐year OS rates between PCa patients versus controls (Δ) were −63% for neuroendocrine versus −16% for signet ring cell versus −6% for ductal and mucinous versus +3% for acinar carcinoma.

### 3.4 OS in metastatic stage PCa patients versus controls

**FIGURE 4:**
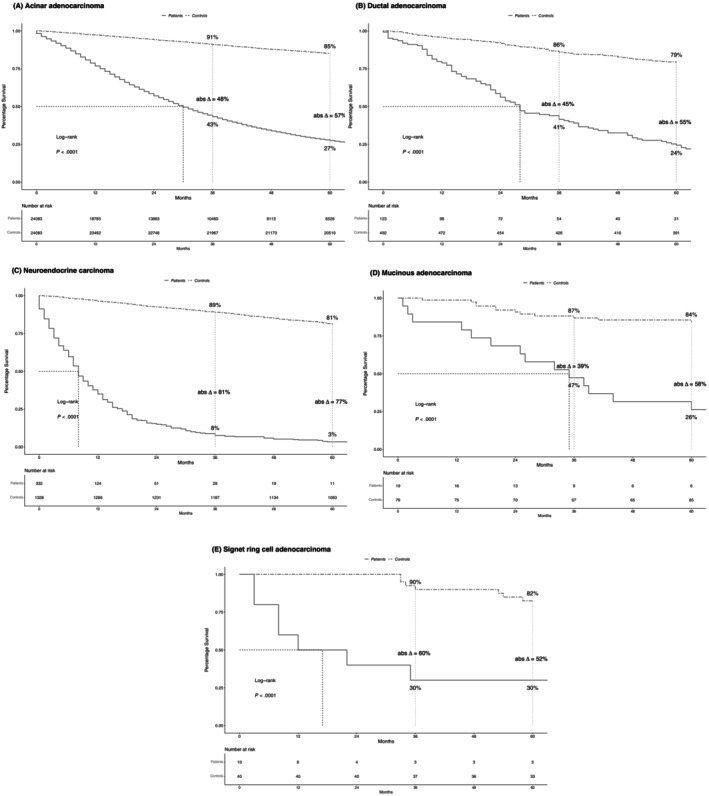
Kaplan–Meier plots comparing overall survival (OS) of patients with metastatic stage (A) acinar, (B) ductal, (C), neuroendocrine, (D) mucinous, (E) signet ring cell carcinoma of the prostate to their simulated age‐matched male population‐based controls. Abs Δ, absolute difference; OS, overall survival.

In metastatic stage, 5‐year OS rates were 3% in neuroendocrine carcinoma, 24% in ductal, 26% in mucinous, 27% in acinar, and 30% in signet ring cell adenocarcinoma patients (Figure 4A–E). In controls that were simulated to match metastatic stage PCa patients, corresponding OS rates were 81%, 79%, 84%, 85%, and 82%, respectively. The resulting differences in 5‐year OS rates between patients versus controls (Δ) were −77% for neuroendocrine versus −58% for mucinous versus −57% for acinar versus −55% for ductal versus −52% for signet ring cell carcinoma.

## 4 DISCUSSION

The magnitude of life expectancy difference between PCa patients harboring rare histological subtypes and age‐matched male population‐based controls is unknown. We addressed this knowledge gap and made several noteworthy observations.

First, histological PCa subtypes other than acinar adenocarcinoma are rare.^1^, ^2^, ^4^, ^5^ Bronkema et al. relied on the National Cancer Database (NCDB) and identified 1958 patients with ductal, 1304 with neuroendocrine, 1020 with mucinous, and 2000 with signet ring cell carcinoma among 1,243,806 PCa patients between 2004 and 2015.^4^ Similarly, Marcus et al. relied on the SEER database and identified 662 patients with ductal, 502 with neuroendocrine, 806 with mucinous, and 130 with signet ring cell carcinoma among 793,064 PCa patients (SEER 1973–2008).^1^ In the current study, we focused on 582,220 PCa patients newly diagnosed between 2004 and 2015 (SEER) and identified 867 ductal, 534 neuroendocrine, 368 mucinous, and 83 signet ring cell carcinoma patients. Single‐ or multi‐institutional series addressing PCa subtypes relied on substantially smaller sample sizes.^16^, ^17^, ^18^, ^19^, ^20^, ^21^, ^22^ Therefore, PCa patients with histological subtypes should ideally be examined in population‐based analyses when life expectancy represents the outcome of interest, as was done in the current study.

Second, we identified important differences in stage distribution between patients with acinar adenocarcinoma versus rare histological PCa subtypes. Within our cohort, metastatic stage was most prevalent in neuroendocrine carcinoma (62%), followed by ductal (14%), signet ring cell (12%), mucinous (5%), and acinar adenocarcinoma (4%). Virtually but not exactly the same rank order was recorded by Bronkema et al. within the NCDB.^4^ However, in that report no comparison was made to age‐matched male population‐based controls. Due to the presence of important differences in stage distribution between the dominant acinar adenocarcinoma and rare histological PCa subtypes, it is essential to rely on stage specific analyses when survival outcomes represent the endpoint of interest, as was done in the current study.

Third, we assessed OS in acinar adenocarcinoma vs. rare histological PCa subtypes in stage‐specific fashion. In stage‐specific analyses, metastatic neuroendocrine carcinoma patients exhibited the worst 5‐year OS rate (3%), followed by locally advanced (16%) and localized stage (22%) neuroendocrine carcinoma patients. Conversely, localized mucinous and locally advanced acinar adenocarcinoma patients exhibited the best 5‐year OS rate (90%). The stage‐specific OS rates of acinar and neuroendocrine patients recorded within the current analysis, validate previous series.^11^, ^12^, ^13^, ^23^ However, to the best of our knowledge, no previous study examined OS rates in ductal, mucinous, and signet ring cell adenocarcinoma patients in stage‐specific fashion. In consequence, no direct comparison with any previous report is possible.

Fourth, relying on United States SSA Life Tables, Monte Carlo simulation methodology and Markov chain of natural progression, we simulated 5‐year OS of controls for all examined histological PCa subtypes and across all stages. Five‐year OS rates ranged from 72% and 77% for controls simulated for purpose of subsequent comparison with localized stage neuroendocrine and ductal carcinoma patients to 90% and 91% for controls simulated for purpose of subsequent comparison with locally advanced stage mucinous and signet ring cell adenocarcinoma patients. In summary, the 5‐year OS rates of simulated controls reflect the age characteristics of PCa patients across different histological subtypes and stages.

Fourth, we compared differences in OS between PCa patients and their controls in stage‐specific fashion. In localized stage, the most pronounced differences in OS at 5 years of follow‐up relative to controls were observed for neuroendocrine carcinoma (Δ −49%), followed by ductal and signet ring cell adenocarcinoma (both Δ −6%). Conversely, no decrease in life expectancy was recorded for mucinous and acinar adenocarcinoma. Similarly, in locally advanced stage, the greatest decrease in life expectancy was recorded in neuroendocrine (Δ −63%), followed by signet ring cell carcinoma (Δ −16%). Conversely, no decrease in OS was recorded for acinar adenocarcinoma. Finally, in metastatic stage, OS decrease (Δ) ranged from 77% for neuroendocrine, to 58% for mucinous, 57% for acinar, 55% for ductal, and 52% for signet ring cell carcinoma patients. These observations do not only indicate that men with newly diagnosed neuroendocrine carcinoma lose a considerable amount of life expectancy at initial diagnosis. They also indicate that neuroendocrine carcinoma patients experience the most pronounced decrease in life expectancy across all examined histological subtypes and stages. Conversely, patients with other rare histological PCa subtypes, such as localized and locally advanced stage mucinous, and ductal adenocarcinoma, experience only a minor decrease in life expectancy. Based on their novelty, the currently reported findings cannot be directly compared with any previous study since no such study addressed rare histological PCa subtypes. Moreover, the above observations indicate that a controlled comparison of metastatic stage ductal (Δ −55%) and acinar adenocarcinoma (Δ −57%) results in virtually the same results regarding life expectancy decrease. Conversely, previous uncontrolled comparison suggested higher mortality rates for metastatic stage ductal compared with acinar adenocarcinoma.^3^ Therefore, these observations further validate the need for controlled comparisons of life expectancy detriments.

Taken together, our observations quantifying differences in 5‐year OS of patients with rare histological PCa subtypes versus age‐matched male population‐based controls are of great clinical and epidemiological importance. The current results provide clinicians with a first head‐to‐head comparison of life expectancy of patients with rare histological PCa subtypes relative to age‐matched male population‐based controls. The reported observations are of great value in counselling and clinical decision making in patients with rare histological PCa subtypes.

In particular, the pronounced decrease in life expectancy across all stages in neuroendocrine carcinoma patients compared with their population‐based controls should encourage clinicians to optimize treatment of these patients. Possible measures to optimize the treatment of patients with neuroendocrine carcinoma of the prostate include the introduction of specialized reference centers for rare histological subtypes of PCa. Moreover, imaging‐guided biopsies, comprehensive pathological evaluation, and molecular testing may provide earlier and more accurate histological diagnosis. Targeted therapies based on molecular profiling, such as immunotherapy (checkpoint inhibitors), should be considered in eligible patients. Last but not least, serum biomarkers such as chromogranin A and neuron‐specific enolase (NSE) should be included in follow‐up of neuroendocrine carcinoma patients. Nevertheless, further research is required to understand the mechanisms underlying neuroendocrine differentiation and to identify new therapeutic targets.

Despite its novelty, the study has limitations. First, the current study shares the limitations of all PCa studies that relied on a retrospective database, such as the SEER^1^, ^2^, ^3^, ^24^, ^25^, ^26^ or the NCDB.^4^, ^8^, ^27^, ^28^ However, SEER or NCDB represent valuable opportunities to study rare cancers with robust statistical conclusions. Second, despite the large scale of the SEER database, the number of patients with rare histological PCa subtypes, especially in metastatic stage, is limited due to the rarity of ductal, neuroendocrine, mucinous, and signet ring cell carcinoma of the prostate. Due to an insufficient number of patients, patients with even rarer histological subtypes of PCa, such as sarcomatoid and adenosquamous carcinoma, could not be included in the present analyses. Third, ICD‐O‐3 histology codes originate from patient records and were not validated by central review. However, the inherent biases associated with this methodology apply to all histological subtypes. Fourth, OS of the control group was derived from United States SSA Life Tables predictions. Although this methodology is well‐established,^11^, ^12^, ^13^, ^14^, ^29^, ^30^, ^31^ it only represents a surrogate for true population‐based controls. Furthermore, United States SSA Life Tables only provide information regarding age and sex. In consequence, adjustments for other patient characteristics, such as race/ethnicity could not be made. Additionally, United States SSA Life Tables derived data do not provide a specific cause of death.^9^ Therefore, the comparison between PCa patients versus controls can only address OS.

## 5 CONCLUSIONS

Regardless of stage, neuroendocrine carcinoma patients exhibit life expectancy that is <50% of that of age‐matched male population‐based controls. The life expectancy detriment in metastatic stage reaches 77%. Conversely, all other rare histological PCa subtypes do not clinically meaningful affect life expectancy in localized or locally advanced stages, except for locally advanced signet ring cell carcinoma where a detriment of 16% was recorded.

## AUTHOR CONTRIBUTIONS

**Carolin Siech:** Conceptualization; methodology; writing – original draft; data curation. **Mario de Angelis:** Conceptualization; writing – review and editing; validation. **Letizia Maria Ippolita Jannello:** Conceptualization; validation; writing – review and editing. **Francesco Di Bello:** Conceptualization; validation; writing – review and editing. **Natali Rodriguez Peñaranda:** Conceptualization; validation; writing – review and editing. **Jordan A. Goyal:** Conceptualization; writing – review and editing; validation. **Zhe Tian:** Conceptualization; methodology; writing – review and editing; data curation. **Fred Saad:** Conceptualization; writing – review and editing; formal analysis. **Shahrokh F. Shariat:** Conceptualization; writing – review and editing; formal analysis. **Stefano Puliatti:** Conceptualization; writing – review and editing; formal analysis. **Nicola Longo:** Conceptualization; writing – review and editing; formal analysis. **Ottavio de Cobelli:** Conceptualization; writing – review and editing; formal analysis. **Alberto Briganti:** Conceptualization; writing – review and editing; formal analysis. **Mike Wenzel:** Conceptualization; writing – review and editing; formal analysis. **Philipp Mandel:** Conceptualization; writing – review and editing; formal analysis. **Luis A. Kluth:** Conceptualization; writing – review and editing; formal analysis. **Felix K. H. Chun:** Conceptualization; writing – review and editing; formal analysis; supervision. **Pierre I. Karakiewicz:** Conceptualization; writing – review and editing; supervision; formal analysis.

## CONFLICT OF INTEREST STATEMENT

The authors certify that there is no conflict of interest with any financial organization regarding the material discussed in the manuscript.

## ETHICS STATEMENT

Due to anonymously coded design of the SEER database, study‐specific Institutional Review Board ethics approval was not required. The study has been conducted in accordance with the principles set in the Helsinki Declaration.

## Data Availability

The data that support the findings of this study are available from the corresponding author upon request.
